# Long-Term Independence of Solar Wind Polytropic Index on Plasma Flow Speed

**DOI:** 10.3390/e20100799

**Published:** 2018-10-17

**Authors:** George Livadiotis

**Affiliations:** Division of Space Science and Engineering, Southwest Research Institute, San Antonio, TX 78238, USA; glivadiotis@swri.edu; Tel.: +1-210-522-3415

**Keywords:** polytropic index, solar wind, entropy, space plasmas

## Abstract

The paper derives the polytropic indices over the last two solar cycles (years 1995–2017) for the solar wind proton plasma near Earth (~1 AU). We use ~92-s datasets of proton plasma moments (speed, density, and temperature), measured from the Solar Wind Experiment instrument onboard Wind spacecraft, to estimate the moving averages of the polytropic index, as well as their weighted means and standard errors as a function of the solar wind speed and the year of measurements. The derived long-term behavior of the polytropic index agrees with the results of other previous methods. In particular, we find that the polytropic index remains quasi-constant with respect to the plasma flow speed, in agreement with earlier analyses of solar wind plasma. It is shown that most of the fluctuations of the polytropic index appear in the fast solar wind. The polytropic index remains quasi-constant, despite the frequent entropic variations. Therefore, on an annual basis, the polytropic index of the solar wind proton plasma near ~1 AU can be considered independent of the plasma flow speed. The estimated all-year weighted mean and its standard error is *γ* = 1.86 ± 0.09.

## 1. Introduction

A polytrope is a thermodynamic process that follows a specific relationship among the thermodynamic variables included in the equation of state, such as, density *n*, temperature *T*, and thermal pressure *P*. Each polytropic relationship indicates a family of streamlines of the fluid or plasma flow. The functional form is, typically, a power-law between two thermodynamic variables, that is,

(1) $$P(\vec{r})=A\cdot n{\left(\vec{r}\right)}^{\;\gamma },\;\mathrm{or}\;n(\vec{r})=B\cdot T{\left(\vec{r}\right)}^{\nu },\;\mathrm{with}\;\nu \equiv 1/(\gamma -1)\;$$

where $n(\vec{r})$, $T(\vec{r})$, and $P(\vec{r})$, are respectively the local density, temperature, and thermal pressure, along the streamline. The polytropic process that follows Equation (1) is a quasi-static change of state in which the specific heat is held constant [1]. The exponent *γ* indicates the polytropic index; *ν* denotes an alternative polytropic index, which defines the effective degrees of freedom $\frac{1}{2}d_{\mathrm{eff}}$ [2]. The polytropic index can be related to the kappa index *κ*_0_ [3,4], that is, the parameter that labels and governs the kappa distributions (Reference [5], Chapter 1). While the polytropic index *γ* (or *ν*) is the same for all the streamlines characterizing the thermodynamic process (although it might be considered to have positional dependence, $\gamma (\vec{r})$ (e.g., Reference [6]), the quantities *A* and *B* are not constants and depend on the certain streamline; thus they do not reduce the dimensionality of the problem, namely, the 2-D thermodynamic space of (*n*, *P*) becomes the 2-D space of (*n*, *A*). They can be substituted by the thermodynamic values at a certain point on the streamline, e.g., $\vec{r}={\vec{r}}_{*}$,

(2) $$[P(\vec{r})/P({\vec{r}}_{*})]={\left[n(\vec{r})/n({\vec{r}}_{*})\right]}^{\;\gamma },\;\mathrm{or}\;[n(\vec{r})/n({\vec{r}}_{*})]={\left[T(\vec{r})/T({\vec{r}}_{*})\right]}^{\nu }\;$$

the values of the polytropic index are characteristic of the thermodynamic process. Starting from the isochoric process for *γ* → −∞, and moving with increasing *γ*, we have the processes of isobaric for *γ* → 0, isothermal for *γ* → 1, adiabatic for *γ* → 5/3, and isochoric again for *γ* → +∞. The four intervals in between correspond to “Explosion” for −∞ < *γ* < 0, “Mild Explosion” for 0 < *γ* < 1, “Sub-adiabatic” for 1 < *γ* < 5/3, and “Super-adiabatic” for 5/3 < *γ* < +∞ (e.g., Reference [5], Chapter 5; References [7,8]). Notes: (i) The polytropic index *a* must not be confused with the ratio of specific heats; their equality holds only in the adiabatic process. (ii) Solar wind has strong fluctuations in magnetic pressure, and plasma beta (ratio of thermal to magnetic pressure); however, the definition and calculation of the polytropic index involves only the thermal pressure (though other types of states have been recently suggested and studied, e.g., References [9,10]). (iii) A minor exception to (ii) is the filtering of data with a constant Bernoulli integral (which depends on the magnetic pressure and other components), for reducing the possibility of streamline crossing.

Analyses of space plasma datasets show that the majority of these plasmas exhibit positive correlations between *n* and *T*, namely, the polytropic index *ν* is positive, thus, *γ* > 1. However, there are several cases of space plasmas with negative correlations between *n* and *T*, i.e., *ν* < 0, in such a way that the polytropic index *γ* is close to zero; this is consistent with constant or quasi-constant thermal pressure. These polytropes were found in the heliosheath [8,11,12,13] and the planetary magnetosheaths, e.g., the low latitude boundary layer at the terrestrial magnetosheath [14]; in the terrestrial central plasma sheet [15,16]; in the Jovian magnetosheath [17]. Rarely, these special polytropes can be also found in the solar wind [18] or the planetary magnetospheres (e.g., Reference [19]).

Nevertheless, the solar wind protons near 1 AU exhibits polytropes with sub-adiabatic indices (1 < *γ* < 5/3), e.g., Totten et al. [20] using Helios-1 data. In particular, References [3,4,21,22] found polytropic indices very close to the adiabatic value, while References [18,23] found that the polytropic index spans a large range of values but the mean is still close to the adiabatic value. Nicolaou et al. [18] used the OMNI database (https://omniweb.gsfc.nasa.gov/) to calculate the polytropic indices of solar wind protons, and found that the distribution of polytropic indices is a *κ*-Gaussian distribution with mean ≈1.8 and standard error of the mean ≈2.4.

The set of polytropic thermodynamic processes for a fixed polytropic index can be extended for a superposition of polytropes. As an example, is the generalization of the equation of state and the Bernoulli’s integral by formulating a superposition of polytropic processes [8]. The superposition is described by a distribution of polytropic indices, but this may be the typical Gaussian distribution. The polytropic density-temperature relationship has been in use of numerous analyses of space plasma data. The linear polytropic relationships on log-log scale are now generalized to concave-downwards parabolas, capable of describing more accurate observations. The model of the Gaussian superposition of polytropa was successfully applied in the inner heliosheath proton plasma. The estimated mean polytropic index is near zero, indicating dominance of isobaric thermodynamic processes in the sheath, similar to other previously published analyses [13].

In this paper, we study the variation of the polytropic index over the last two solar cycles (years 1995–2017) for the solar wind proton plasma near Earth (~1 AU). The paper is organized as follows. In Section 2, we describe the datasets used in this study. In Section 3 we describe the methodology for determining the polytropic index (time series, weighted means and their standard errors, 2-D histograms). In Section 4 we present the results of the derived time series of polytropic indices, as well as their weighted means and standard errors per selected bin of the solar wind speed and per year. We summarize our findings in Section 5.

## 2. Datasets

**Figure 1:**
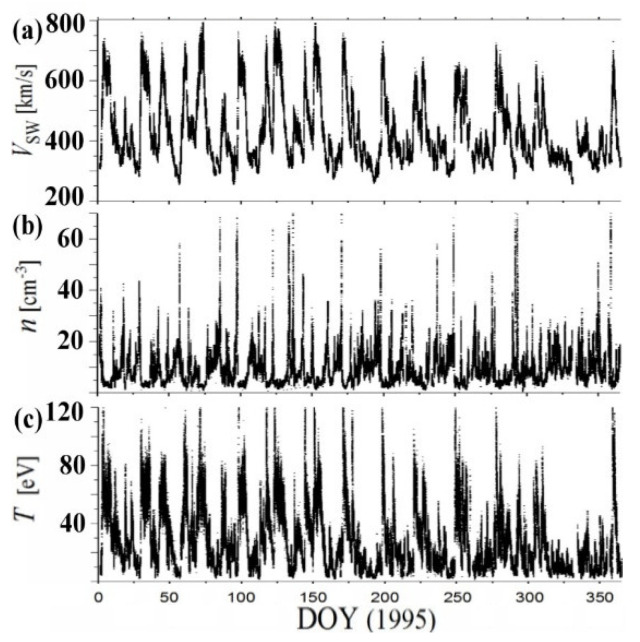
Data used in this paper: ~92-s resolution measurements of bulk solar wind plasma moments (**a**) speed *V*_sw_; (**b**) density *n*; and (**c**) temperature *T*, recorded from SWE onboard Wind S/C, during the year 1995.

We calculated the polytropic index using high-resolution (~92-s averaged) data of solar wind proton plasma moments (speed *V*_sw_, density *n*, and temperature *T*), e.g., References [24,25,26,27,28]; see also Reference [4], near ~1 AU, as measured from the Solar Wind Experiment (SWE) instrument onboard Wind spacecraft (S/C), publicly accessible at the mission database (https://wind.nasa.gov/data.php), or at the OMNIWeb-Plus database, which includes the solar wind phase only (https://omniweb.gsfc.nasa.gov/ftpbrowser/wind_swe_2m.html). We repeated our calculations for all the years in the period 1995–2017, spanning the two solar cycles 23 and 24 (Figure 1). Notes: (1) Wind is a spin-stabilized S/C launched on 1 November 1994. After several orbits through the Earth’s magnetosphere, Wind was placed in early 2004 in a halo orbit around the L1 Lagrange point. (2) The distinct periodicities characterizing the solar wind plasma moments, e.g., 5 day, 13.5 day, 45 day, etc., were shown by wavelet spectrum and other statistical analyses, e.g., References [29,30,31,32].

## 3. Methods

We used the density *n* and temperature *T* of the *i*th and (*i* + 1)th data points, to derive the polytropic index at the *i*th data point given by:

(3a) $$\gamma_{i}=1+\ln(T_{i+1}/T_{i})/\ln(n_{i+1}/n_{i})\;$$

with error given by propagation, i.e.,

(3b) $$\begin{array}{l} \delta \gamma_{i}=\sqrt{2}\cdot \sqrt{{\left(\delta \ln T\right)}^{2}+{\left(\gamma_{i}-1\right)}^{2}{\left(\delta \ln n\right)}^{2}}\cdot {\left[\ln(n_{i+1}/n_{i})\right]}^{-1}\\ =0.113\cdot \sqrt{1+0.141\;{\left(\gamma_{i}-1\right)}^{2}}\cdot {\left[\ln(n_{i+1}/n_{i})\right]}^{-1}\;, \end{array}$$

originated by the propagation of temperature δlnT=δT/T≅8% and density δlnn=δn/n≅3% errors (log-normally distributed; for Wind SWE data, see [25]). Note: We apply Equation (3a) only when *i*th and (*i* + 1)th data points correspond to invariant Bernoulli integral, in order to reduce the possibility of streamline crossing [18,23].

The *M-step* moving average is given by (according to Reference [22]):

(4a) $$\bar{\gamma }(M)=\sum_{i=1}^{M}\delta \gamma_{i}{}^{-2}\gamma_{i}/\sum_{i=1}^{M}\delta \gamma_{i}{}^{-2}\;$$

The error of the weighted mean is a combination of the propagated uncertainty:

(4b) $$\delta {\bar{\gamma }}_{}^{prop}=\frac{1}{\sqrt{\sum_{i=1}^{M}\delta \gamma_{i}^{-2}}}\;$$

and the statistical uncertainty (e.g., References [33,34]):

(4c) $$\delta {\bar{\gamma }}_{}^{stat}=\sqrt{\frac{\frac{1}{M}\sum_{i=1}^{M}\delta \gamma_{i}^{-2}{\left(\gamma_{i}^{}-\bar{\gamma }\right)}^{2}}{\sum_{i=1}^{M}\delta \gamma_{i}^{-2}-{\left(\sum_{i=1}^{M}\delta \gamma_{i}^{-2}\right)}^{-1}\cdot \sum_{i=1}^{M}\delta \gamma_{i}^{-4}}}\;$$

which are combined to give the total uncertainty of the weighted mean:

(4d) $$\delta \bar{\gamma }(M)=\sqrt{{\left(\delta {\bar{\gamma }}_{}^{prop}\right)}^{2}+{\left(\delta {\bar{\gamma }}_{}^{stat}\right)}^{2}}\;$$

(For several applications of the above statistics, see: References [34,35,36,37,38,39]).

**Figure 2:**
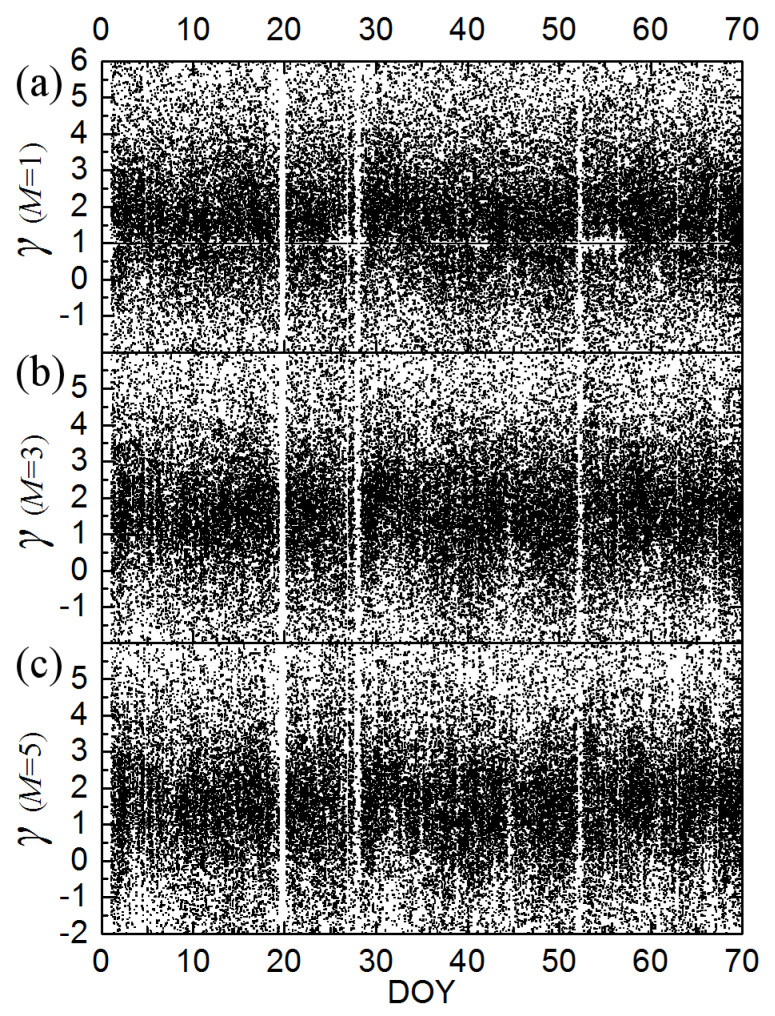
Polytropic indices calculated using (**a**) *M* = 1; (**b**) *M* = 3; and (**c**) *M* = 5 consecutive values of (*n*, *T*) from ~92-s resolution measurements of the bulk solar wind plasma parameters from the SWE instrument onboard Wind during the year 1995. (Note: The white stripes are caused either by lack of solar wind data observations, or by highly erroneous collected data which have been neglected from our statistical analysis.).

Using Equations (3a) and (3b) and Equations (4a)–(4d), we derived the polytropic index *γ* and its moving average *γ_M_* for step *M* = 1–5, and their errors. We constructed the time-series for *γ_M_*, for *M* = 1, 3, 5, for the first 70 days of 1995, plotted in Figure 2. (Obviously, the case of *M* = 1 corresponds to the raw time series of *γ* values).

**Figure 3:**
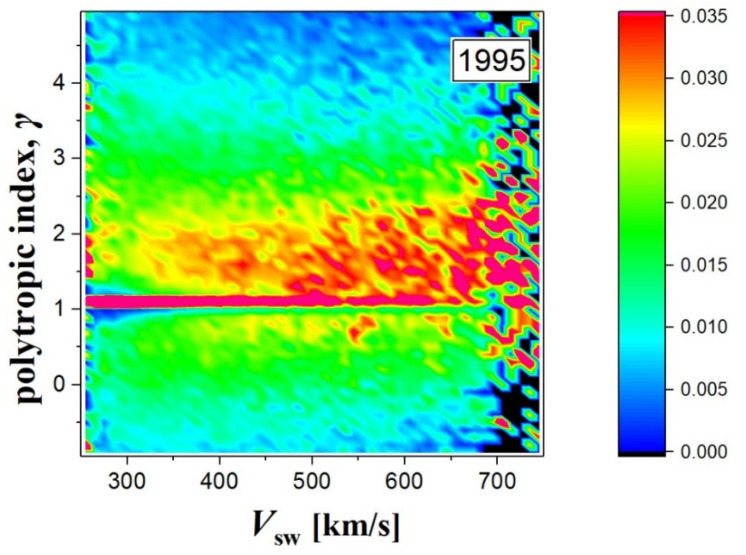
2D-histogram (or occurrence frequency) of the values of the speed *V*_sw_ and polytropic index *γ_M_* with *M* = 5 (plotted in Figure 2c) of the solar wind proton plasma, observed during the year 1995.

We, then, constructed the normalized 2D-histograms of (*V*_sw_, *γ_M_*) for *M* = 5 moving averages to the data from Figure 2a. Increasing the step *M* stabilizes the 2D-histogram, as it does in this case for *M* = 5. We normalized the 2D-histograms to investigate the actual relationship between *γ* and *V*_SW_. Figure 3 shows the 2D-histogram normalized by the 1D-histogram of *V*_SW_, which clearly demonstrates that the distribution of polytropic indices has weak dependence of the solar wind speed, at least for the year 1995.

**Figure 4:**
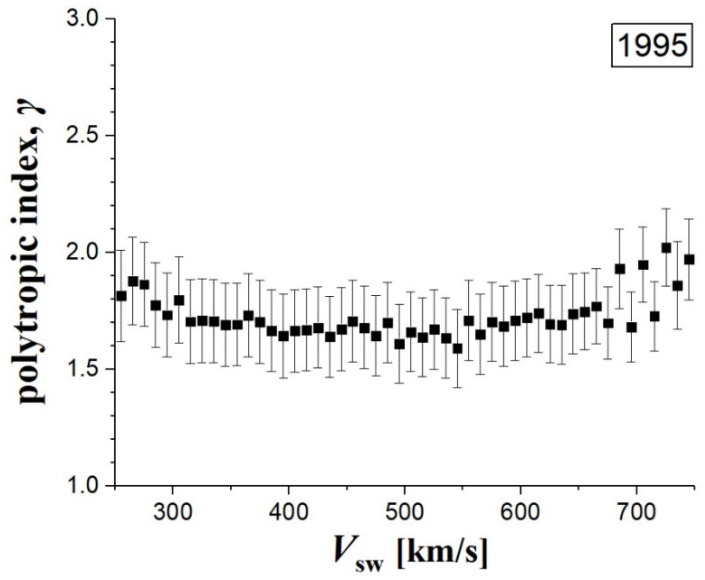
Mean and standard error of the polytropic indices *γ_M_* with *M* = 5, estimated for each *V*_SW_-bin.

This weak dependence of polytropic indices with respect to *V*_SW_ is also shown in Figure 4. This figure shows the mean values and the standard error of the mean, for the polytropic indices, estimated for each of the *V*_SW_-bins. The polytropic index appears weakly increased for the lower and higher speed values. The weighted mean value of the polytropic index from all the bins in 1995 is ≈ 1.69 ± 0.03, which is consistent with previously published estimations of this index in the solar wind near 1 AU during 1995 (e.g., Reference [22]).

## 4. Results

Here, we estimated the average values and errors of the polytropic indices. In particular, we first determined the polytropic indices that characterized the solar wind plasma observed over two solar activity cycles at 1 AU. Namely, we generated the datasets of polytropic index for the raw data of the yearly time-series (1995–2017). Then, we estimated the weighted means and errors for each bin of the solar wind speed and for each year (1995–2017).

**Figure 5:**
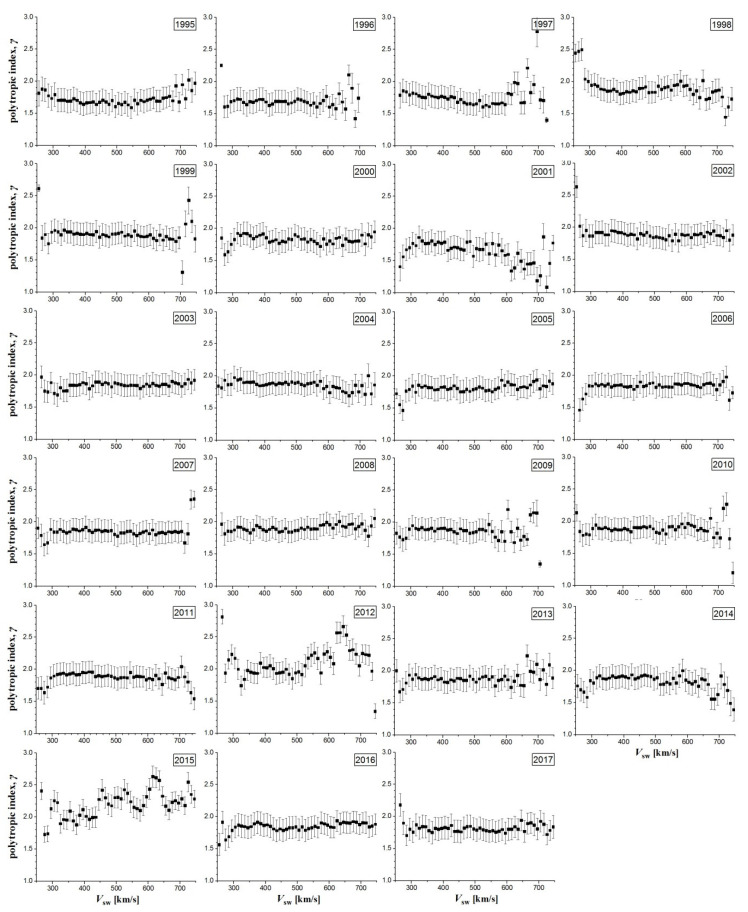
Polytropic indices (weighted means and errors) of solar wind plasma at 1 AU (1995–2017).

Figure 5 plots all the graphs similar to that of Figure 4, that is, the polytropic indices (weighted means and errors) of the solar wind plasma at 1 AU, for each year from 1995 to 2017, spanning two solar cycles. The polytropic index appears to remain quite constant with the solar wind speed for most of the annual graphs, exhibiting its fluctuations usually in the fast solar wind, i.e., for *V*_sw_ > 550 km/s (that is a typical separatrix between the two solar wind modes).

This quasi-constancy of the solar wind polytropes to the plasma flow speed agrees with the results of Totten et al. [20]. These authors determined the polytropic index using proton data from Helios-1 S/C, where they estimated an average value of *γ* ≈ 1.46; when the magnetic pressure was also included (together with the thermal pressure) they found *γ* ≈ 1.58. For the year 1995, we find *γ* ≈ 1.69 ± 0.03 (Table 1), while for the first 70 days of that year the polytropic index is *γ* ≈ 1.63 ± 0.05 [22]. (There is a chance that the polytropic index decreases with the heliocentric distance *r*_s_, since the Helios-1 datasets span *r*_s_ from ~0.3AU to ~1 AU). What is most important to mention is that Reference [20] found the polytropic index to be independent of the plasma flow speed. The same result was shown in Reference [22] for the solar wind plasma near ~1 AU.

**Figure 6:**
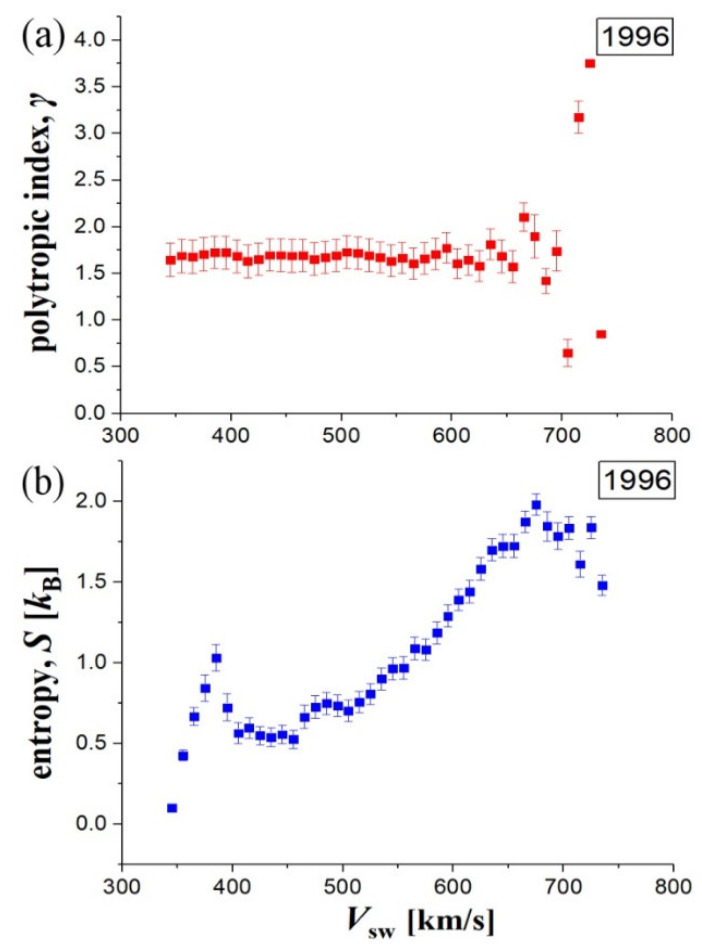
(**a**) Polytropic index *γ* (red), and (**b**) entropy *S* (associated with kappa distributions) (blue), characterizing the solar wind proton plasma ~1 AU, plotted using Wind S/C data for the year 1996.

The constancy of the polytropic index is independent of the fact that the solar wind proton plasma has significant variations in the bulk parameters (e.g., see Reference [4]). We also calculated the entropy of the plasma, which is formulated in terms of temperature and kappa index (Reference [5], Chapter 2; References [40,41,42]). We observed that even the entropy appears to change significantly with the solar wind speed, while the polytropic index remains quasi-constant (Figure 6). The polytropic index has no reason to exhibit a significant (average) variation, e.g., the same thermodynamic processes characterize the solar wind plasma; however, the entropy tends to increase with solar wind speed. This is observed, mainly because the temperature increases with speed in the solar wind plasma [43]. The latter behavior may be explained by several phenomena, such as the dispersion of magnetosonic waves [22]. Notes: (i) Any possible fluctuations on solar wind bulk parameters will generally affect more the entropy rather than the polytropic index estimations, because the latter is derived from the differences of those parameters. (ii) The local maximum of entropy for speed *V*_sw_ ~390 km/s may reflect a physical reasoning, that is, an increase of solar wind plasma particles fluctuations; however, it may be an artifact caused by erroneous datasets collected along the slow solar wind (e.g., the same reason may cause the slow decreasing of polytropic indices in Figure 4).

**Figure 7:**
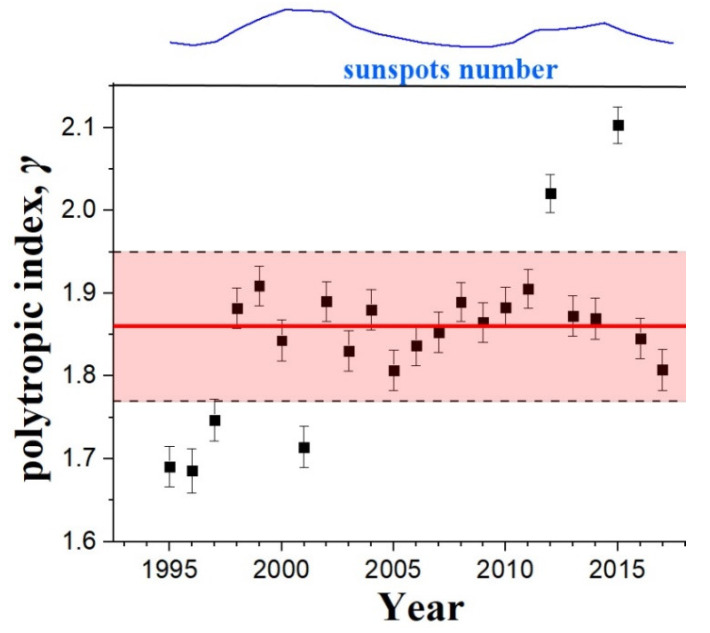
Annual average values of the polytropic indices and their standard errors of the mean. The red-shaded rectangular indicates the estimated all-year weighted mean (red solid) and its standard error, that is, *γ* = 1.86 ± 0.09.

Table 1 shows the annual mean values and standard errors of the mean, for each year from 1995 to 2017, that is, for the two solar cycles 23 and 24. Figure 7 shows the annual average values of the polytropic indices and their standard errors of the mean. There is no clear correlation with the yearly sunspots number. The estimated all-year weighted mean and its error is *γ* = 1.86 ± 0.09 (shown in the figure within the transparent red rectangular), corresponding to 2.3 effective degrees of freedom (super-adiabatic process). This derived long-term value of the polytropic index agrees with the results of Reference [18].

**Figure 8:**
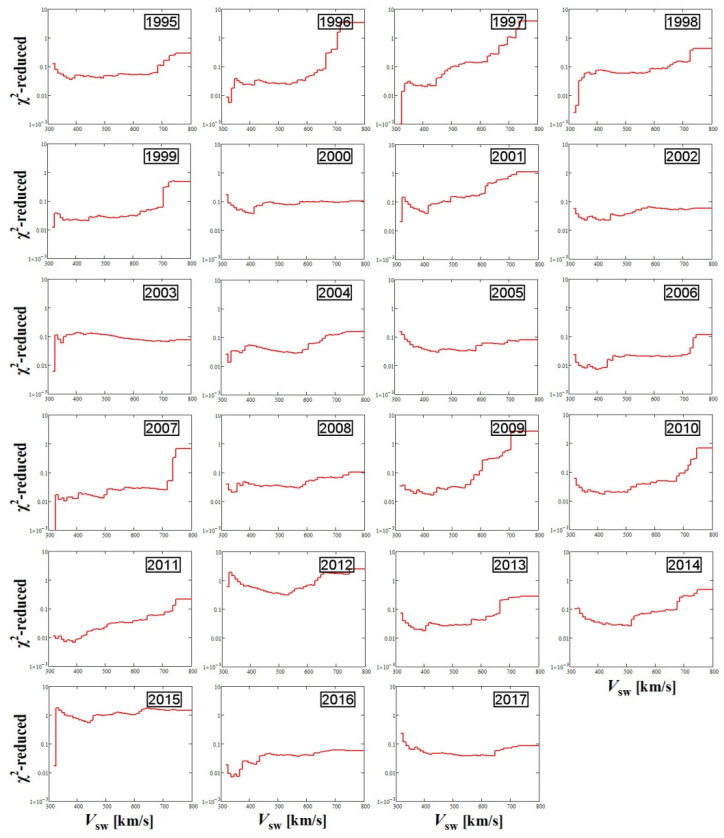
Reduced chi-square values (1995–2017), derived from fitting the annual plots of the polytropic indices with the plasma flow speed (Figure 5) with a constant.

**Figure 9:**
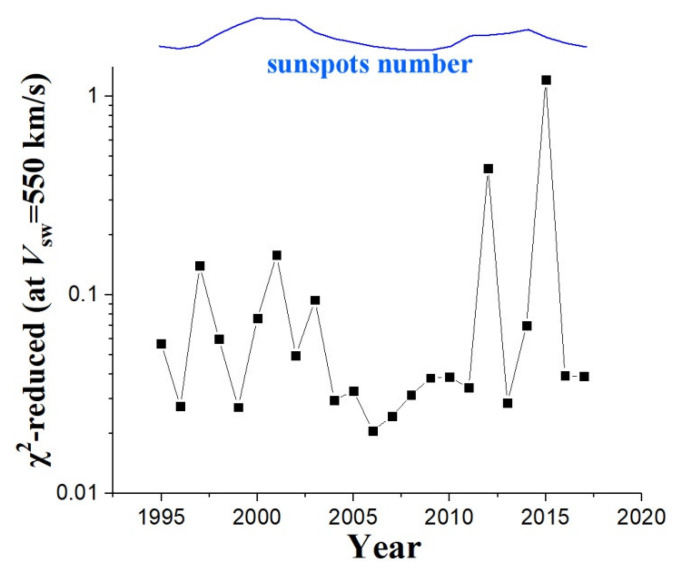
Reduced chi-square values (1995–2017), for plasma flow speed *V*_sw_ = 550 km/s (Figure 8).

As we mentioned, the polytropic index appears to fluctuate and departs from the quasi-constancy mostly in the fast solar wind, i.e., typically for *V*_sw_ > 550 km/s. This becomes clearer in Figure 8, where we depict the plots of the reduced chi-square values (1995–2017), which are derived from fitting the annual plots of the polytropic indices with the plasma flow speed (in Figure 5) with a constant:

(5) $$\chi_{\mathrm{red}}^{2}=\frac{1}{N-1}\sum_{i=1}^{N}\delta \gamma_{i}^{-2}{\left(\gamma_{i}^{}-\bar{\gamma }\right)}^{2}\;$$

where *N* = 50 is the number of plasma flow speed bins. The reduced chi-square values, taken for plasma flow speed *V*_sw_ = 550 km/s for each year, is shown in Figure 9 (the plot of sunspots number is also depicted for comparison). We observe a disturbed chi-square value at the two solar maxima and a smooth value at solar minimum.

The above results suggest that it is rather more possible for high velocities of the solar wind plasma to develop intermittent turbulent states [44]. In addition, the kappa indices may be in correlation with the polytropic indices [3], in general, so that the study of polytropes to direct to non-extensive statistics. Nevertheless, the research on this topic is still at an early stage.

## 5. Conclusions

We derived the polytropic indices over the last two solar cycles (years 1995–2017) for the solar wind proton plasma near Earth (~1 AU), using high-resolution ~92-s datasets of proton plasma moments (speed *V*_sw_, density *n*, and temperature *T*), measured from the SWE instrument onboard Wind S/C.

The polytropic index was estimated for five point moving averages. We constructed the time series of the derived polytropic indices, as well as their weighted means and standard errors per selected bin of the solar wind speed and per year. The derived long-term behavior of the polytropic index agrees with the results of other previous methods.

The main results are summarized as follows:

- The polytropic index remains quasi-constant with respect to the plasma flow speed. This result agrees with the results of previous analyses of solar wind plasma, even for smaller heliocentric distances.
- The polytropic index remains quasi-constant, despite the frequent entropic variations.
- Most of the fluctuations of the polytropic index appear in the fast solar wind.
- The estimated all-year weighted mean and its standard error is *γ* = 1.86 ± 0.09.

Therefore, on an annual basis, the polytropic index of the solar wind proton plasma near ~1 AU can be considered independent of the plasma flow speed.

## Figures and Tables

**Table 1:** Annual average values of the polytropic indices and their standard errors of the mean.

Year	$\bar{\gamma }(M=5)\;$	$\delta \bar{\gamma }(M=5)\;$
1995	1.690614	0.024779
1996	1.685573	0.026863
1997	1.747082	0.025609
1998	1.882041	0.024076
1999	1.908898	0.023941
2000	1.843102	0.02476
2001	1.714217	0.024866
2002	1.890045	0.024366
2003	1.830606	0.024548
2004	1.879968	0.024253
2005	1.807218	0.024583
2006	1.837272	0.024523
2007	1.853058	0.024455
2008	1.889664	0.023458
2009	1.864729	0.024021
2010	1.88307	0.024246
2011	1.905485	0.023795
2012	2.020513	0.022979
2013	1.872648	0.024541
2014	1.869276	0.02453
2015	2.103356	0.022067
2016	1.845605	0.024372
2017	1.807524	0.024706
All-Years Average	1.86	0.09
